# Effects of UV Radiation on the Chlorophyte *Micromonas polaris* Host–Virus Interactions and MpoV-45T Virus Infectivity

**DOI:** 10.3390/microorganisms9122429

**Published:** 2021-11-25

**Authors:** Charlotte Eich, Sven B. E. H. Pont, Corina P. D. Brussaard

**Affiliations:** 1 Department of Marine Microbiology and Biogeochemistry, NIOZ Royal Netherlands Institute for Sea Research, 1797 SZ t’Horntje, The Netherlands; sven.pont@nioz.nl; 2 Institute for Biodiversity and Ecosystem Dynamics (IBED), University of Amsterdam, 1098 XH Amsterdam, The Netherlands

**Keywords:** arctic, *Micromonas polaris*, UV-AB, UV-C, virus decay, virus production

## Abstract

Polar seas are under threat of enhanced UV-radiation as well as increasing shipping activities. Considering the ecological importance of marine viruses, it is timely to study the impact of UV-AB on Arctic phytoplankton host–virus interactions and also test the efficacy of ballast water (BW) UV-C treatment on virus infectivity. This study examined the effects of: (i) ecologically relevant doses of UV-AB radiation on *Micromonas polaris* RCC2258 and its virus MpoV-45T, and (ii) UV-C radiation (doses 25–800 mJ cm^−2^) on MpoV-45T and other temperate algal viruses. Total UV-AB exposure was 6, 12, 28 and 48 h (during the light periods, over 72 h total). Strongest reduction in algal growth and photosynthetic efficiency occurred for 28 and 48 h UV-AB treatments, and consequently the virus production rates and burst sizes were reduced by more than half (compared with PAR-only controls). For the shorter UV-AB exposed cultures, negative effects by UV (especially Fv/Fm) were overcome without impacting virus proliferation. To obtain the BW desired log^−4^ reduction in virus infectivity, a UV-C dose of at least 400 mJ cm^−2^ was needed for MpoV-45T and the temperate algal viruses. This is higher than the commonly used dose of 300 mJ cm^−2^ in BW treatment.

## 1. Introduction

Phytoplankton depend on photosynthetic active radiation (PAR) for their primary production, but are also exposed to UV radiation in the surface waters of the ocean [1]. Natural UV-radiation reaching the Earth’s surface is a combination of UV-A (315–400 nm) and UV-B (280–315 nm), while UV-C (100–280 nm) is largely absorbed in the Earth’s atmosphere [2]. The exact concentration of light (both PAR and UV) that phytoplankton in the surface ocean experience, depends on atmospheric ozone depletion, vertical mixing, ice cover, cloud cover and the turbidity of the water [2,3,4]. Especially in polar waters, the UV dose in summer is relatively high and may impact phytoplankton to a large extent [5,6]. Daily atmospheric UV doses in summer for the Arctic are on average 6.6 W m^−2^ for UV-A and 0.4 W m^−2^ for UV-B [7,8], and for the Southern Ocean 27.9 W m^−2^ for UV-A and 1.1 for UV-B [9,10,11]. Average mixed layer UV doses are 1.2 W m^−2^ and 0.06 W m^−2^ for UV-A and UV-B in the Southern Ocean, and 0.23 W m^−2^ and 0.04 W m^−2^ for UV-A and UV-B in the Arctic Ocean, respectively (Supplement Table S1; based on [7,8,9,10,11,12,13]). There is a seasonal loss of stratospheric ozone in the Southern Ocean, leading to an increased exposure to biologically harmful UV-B radiation [6]. In the Arctic, the formation of so called ozone holes are more variable, increasing by 10% from 1983 to 2003 [14] and reaching a new maximum in 2020 [15]. These polar ozone depletions occur at the same time as phytoplankton blooms begin [16], and are estimated to decrease annual primary production from 0.25% [17] up to 12% [18]. With climate change, the UV intensity is predicted to increase for the polar regions. Predicted strengthening of vertical stratification resulting from ice melt and warming can lead to additional UV exposure [19]. Conversely, the input of coloured dissolved organic matter into the Arctic Ocean is predicted to increase, potentially causing a shading effect from UV radiation [20].

UV radiation is generally reported to cause photo-inhibition in phytoplankton, and reduce primary production and growth, although enhanced phytoplankton carbon fixation and (temperature dependent) repair of UV-B induced DNA damage was found under low (≤4.12 W m^−2^) or fast-fluctuating solar irradiance [21,22,23,24,25]. Other marine organisms are also adversely affected by UV irradiation, including heterotrophic bacteria [6,26]. Furthermore, natural UV radiation is an important factor for the loss of viral infectivity and virus decay in the surface ocean [27,28,29]. The ecological consequences of UV-impacted microbial host and virus activity are still largely unknown, despite the increasing awareness that viruses are an important mortality factor for polar microorganisms [30,31,32,33]. The one study using eukaryotic phytoplankton and their viruses showed virus-specific responses to UV-B while UV-A had no effect on virus activity [34]. Experiments providing insight in virus proliferation are, to the authors’ knowledge, not yet reported.

Due to global climate change the Arctic seas and oceans are warming, resulting in earlier and strengthened vertical stratification [35]. Picoeukaryotic phytoplankton such as *Micromonas* (Chlorophyta, Mamiellophyceae) have been reported and predicted to dominate the phytoplankton community under these conditions [36,37,38]. *Micromonas* is readily infected by viruses [39,40,41], and in 2017 Maat et al. brought the first viruses infecting an Arctic protist into culture. The production of these *M. polaris* viruses (MpoVs) is sensitive to light availability and temperature [42]. The aim of the current study was to examine the effects of natural doses of UV-AB radiation on the host–virus interactions using the polar model system *Micromonas polaris* RCC2258 and its virus MpoV-45T.

Additionally, the effect of UV-C radiation on virus infectivity was tested using this polar host–virus model system (and compared with temperate algal viruses). With the increasing ice melt in the Arctic, it can be anticipated that shipping will intensify in the very near future (sea routes will be navigable for longer for ships without ice-breaking hulls, moreover new sea routes might become ice-free) [43,44], increasing the possibility of introducing invasive microbes. Invasive species and viruses are considered major threats to biodiversity [45,46]. To reduce (and ideally prevent) the spread of organisms, ships in international traffic need to treat their ship’s ballast water (BW) before discharge [47]. UV-C is the most commonly used disinfection treatment of BW, as it is cheap and environmentally safe as no toxic compounds are discharged [48,49,50,51]. With growing maritime activity, more Artic water will be used as ballast water, but it is as yet largely unclear if the currently used UV doses for BW treatment work effectively for polar viruses. The sensitivity of algal viruses to UV-C is also reported in this work.

## 2. Materials and Methods

### 2.1. UV-AB Experimental Design

To test the effect of UV-AB, the phytoplankton host–virus model system *Micromonas polaris* strain RCC2258 (Roscoff Culture Collection, Roscoff, France) with its virus MpoV-45T [52] was used. This lytic dsDNA virus [52] specifically infects *M. polaris*, resulting in full lysis of the host culture. The phytoplankton species were grown in Mix-TX medium [53] under 40–60 µmol quanta m^−2^ s^−1^ PAR (ca 8.6–13 W m^−2^ [54]) (TLD 90 DeluxePro 18W/965 or MASTER TL-D 90 De Luxe 36W/965, Philips, Eindhoven, The Netherlands) and a light–dark cycle of 16:8 h. Cultivation temperature was 3 °C for the polar and 15 °C for the temperate virus–host systems. All phytoplankton cultures were kept in exponential growth phase. Virus cultures were maintained by infecting the specific host with a 10% *v*/*v* addition of the viral lysate. Infected cultures were checked for host cell lysis by eye (clearance of the cultures). Viral lysis of the host cells is due to the release of the virus progeny and typically starts 16–24 h after infection and full lysis occurs around 80–100 h [52]. These experiments showed typical host abundance dynamics upon infection [52].

### 2.2. Enumeration of Phytoplankton and Viruses

Phytoplankton were counted fresh using a Beckton Dickinson FACSCalibur flow cytometer with a 488 nm argon laser [55], with the trigger on chlorophyll red autofluorescence. Viruses were enumerated according to the protocol by Brussaard et al. [56] with modifications by Mojica et al. [57]. In short, virus samples were fixed with glutaraldehyde (EM-grade, 0.5% final concentration; Sigma-Aldrich, St. Louis, MO, USA) for 15–30 min at 4 °C, after which the samples were flash-frozen in liquid N_2_ and stored at −80 °C until analysis. After thawing the samples, they were diluted with Tris-EDTA buffer (1M Tris, 0.5M Na_2_-EDTA, pH of 8.2), stained with nucleic acid-specific SYBR Green I (0.5 × 10^−4^ concentration of the commercial stock, Invitrogen Molecular Probes, Eugene, OR, USA) at 80 °C for 10 min in the dark. The trigger was set to green fluorescence. FCS express 5 (De Novo Software, Pasadena, CA, USA) was used for all flow cytometric data analyses, with gating performed on red chlorophyll autofluorescence vs. side scatter for the phytoplankton and SYBR Green fluorescence vs. side scatter for the viruses.

### 2.3. UV-AB Experiment

Phytoplankton cultures were grown under a 16:8 h light–dark cycle (PAR intensity as described earlier) and were exposed to UV-AB radiation during the light periods for a total of 6, 12, 28 and 48 h. This means that, e.g., the measurements for 28 h UV-treatment took place after 36 h (28 h UV+PAR and 8 h darkness). The light–dark cycle can allow for (partial) damage repair, which might mitigate the potential UV-AB effects. Even during 24 h polar days, however, the UV radiation received in the early and late hours of the day is low [58]. The UV exposure time was chosen so that UV was applied throughout the MpoV-45T latent period (16–20 h at PAR-only culture conditions [42,52]), and before the host cells lysed. Cultures were not stirred as prior tests determining the growth of cultures mixed at 100 rpm (magnetic stirrer) and those mixed gently only when sampled showed that the mixed cultures showed reduced growth by more than half, compared with the non-stirred culture (over the 72 h of the experimental incubation time).

One-step infection experiments were performed with freshly prepared MpoV-45T viral lysate that was added to exponentially growing *M. polaris* cultures (5.3–7.6 × 10^5^ mL^−1^ starting abundance) 2–3 h into the light period, at a virus to host cell ratio of around 20. UV radiation was switched on at the same time viruses were added. There were controls for both the UV treatment (PAR-only) and viral infection (non-infected). Non-infected controls were checked, and no viruses were detected. The UV-AB experiment was divided in two different experiments as the number of quartz Erlenmeyer flasks was limited: UV-AB experiment 1 (0, 6, 12, 48 h exposure) and UV-AB experiment 2 (0, 28 and 48 h exposure). Experiments were performed in duplicate, except for PAR-only control of UV-AB experiment 2. Samples for algal host and virus enumeration were taken at regular time intervals during the first 24 h, followed by less frequent sampling until 72 h p.i. Viral production rates (h^−1^) were estimated from 21–25 h to 71–72 h after infection (based on the latent period, i.e., time until progeny viruses are released from host cells, of MpoV-45T being 16–24 h [52]). Viral burst size, defined as the number of viruses released per lysed host cell, were calculated by dividing the number of viruses produced by the number of host cells lost (maximum minus minimum count for both the numerator and denominator).

### 2.4. Specific Algal Growth Rates and Photosynthetic Efficiency

During the experiments, the specific exponential growth rates (d^−1^) of the (non-infected) *M. polaris* cultures were determined from the temporal abundance dynamics. For 6–28 h UV-treated cultures, only the period after UV exposure was used for the estimations. For 48 h UV and PAR treatments, rates were calculated from t0 onwards, with the exception of PAR-controls from experiment 2, as phytoplankton abundance initially declined until t10, thus here growth rates were calculated from t10 onwards. The photosynthetic efficiency (Fv/Fm) of *M. polaris* was determined during the UV exposure experiments at similar time points as abundance samples were obtained. The 3.5 mL algal samples (gently poured into a Water-K quartz cuvette) were dark-adapted for 1 h at incubation temperature (no significant differences in Fv/Fm between 15, 30 and 60 min dark adaptation for *M. polaris*), followed by measuring the minimum fluorescence (Fo) using pulse amplitude modulated fluorometry (Heinz Walz WATER-PAM Chlorophyll Fluorometer Control unit (S/N UKEA0117) with Red LED WATER-ED cuvette version (S/N EDEE0196); software package WinControl-3.21, all from Heinz Walz, Effeltrich, Germany). The maximum fluorescence (Fm) was measured after a saturation pulse (measuring light 3 LEDs peaking at 730 nm; saturation pulse 12 LEDs peaking at 660 nm; maximum saturation pulse density 4000 µmol quanta m^−2^ s^−1^; pulse duration 0.8 s; photon flux density of measuring light <0.1 µmol quanta m^−2^ s^−1^). Fv/Fm was obtained using the following formula: Fv/Fm = (Fm − Fo)/Fm. The maintained algal cultures typically had Fv/Fm values of around 0.5.

### 2.5. UV-AB Doses

The UV-AB lamp (Q-panel Lab Products UV-A-340, Westlake, Canada) produced a UV spectrum very similar to the solar spectrum (measured with aRAMSES ACC radiomenter, TriOS, Rastede, Germany). UV doses were measured using an ULM-500 universal light meter (Heinz Walz GmbH D-91090, Effeltrich, Germany). The following UV intensities were applied: 0.57 W m^−2^ for UV-A and 0.03 W m^−2^ for UV-B, corresponding to maximum simulated depths of 9–16 m for the Arctic and 14–26 m for the Southern Ocean. The total UV-AB doses (over the 72 h of the experiment) applied were: 12,886, 25,772, 60,137 and 103,092 W m^−2^. All simulated depths lay within the average mixed layer depth of both the Arctic in summer (30 m; [59]) and Antarctic waters (50 m; [60]). UV intensities were corrected for intensity reduction by the quartz glass Erlenmeyers and phytoplankton concentrations. The simulated depths were calculated using the following formula:

(1) $$D_{S}=\ln\frac{\mathrm{MD}}{I_{S}\times {\;µ}_{\mathrm{ac}}}$$

where D_S_ stands for simulated depth (m), MD for measured UV intensity (W m^−2^), I_S_ for UV irradiance in air (W m^−2^), and µ_ac_ for attenuation coefficient (m^−1^). To determine the maximum depth simulated, maximum measured UV irradiances in air and lowest attenuation coefficients were used. For the Arctic, used values were 19 W m^−2^ and 1.09 W m^−2^ UV-A and UV-B, respectively [8], and for the Antarctic, 35 W m^−2^ [9] and 1.8 W m^−2^ [11] for UV-A and UV-B, respectively. Attenuation coefficients used for UV-A and UV-radiation in the Arctic were 0.39 m^−1^ and 0.225 m^−1^, respectively [11], and in the Southern Ocean, 0.157 m^−1^ and 0.284 m^−1^, respectively [13]. UV-intensities measured were 0.57 W m^−2^ (UV-A) and 0.03 W m^−2^ (UV-B).

### 2.6. UV-C Inactivation Experiment

The effect of UV-C (irradiances of 0.64 to 0.67 W m^−2^ (0.64 to 0.67 mJ cm^−2^ s^−1^; UV-C emitted by a Hanovia low pressure (LP) lamp with Atlantic ultraviolet UV-C 15260-L40, emitting at 254 nm, Fairfield, NJ, USA) was tested on MpoV-45T. Additionally, two other non-polar nucleocytoplasmic large dsDNA viruses (NCLDV) were used as a comparison to contextualise the effect of UV-C on the infectivity of MpoV-45T, specifically acknowledging the lack of information as to what extent UV-C treatment affects polar viruses. The virus MpV-08T [61] infects another *Micromonas* host species (*M. commoda*, formerly known as *M. pusilla* [62], strain LAC38, Marine Research Center culture collection, Göteborg University) originating from temperate waters [61], whereas the virus PgV-07T [63,64] infects a non-related algal species *Phaeocystis globosa* (strain G(A), Haptophyta, Prymnesiophyceae; Culture collection University of Groningen, The Netherlands) occurring in similar temperate waters [63,64]. Host and virus growth and infection conditions were the same as for the UV-AB experiment.

The UV-C doses were chosen based on a pilot test (C.P.D.B. unpublished results) and the dose applied in a type approval process for ballast water treatment (300 mJ cm^−2^, [65]). UV-C doses were controlled by exposure time and final UV-C doses received were 0 (control), 25, 50, 100, 200, 400, and 800 mJ cm^−2^. Exposure time was 0 s for the control and between 38 and 1213 s and was calculated following Bolton and Linden ([66], see Table S2 for details). Viral infectivity was determined with an end-point dilution assay [67], using the MPN Assay Analyzer [68]. The fraction of infective viruses was calculated by dividing the MPN score with the total viral abundance. MPN assays were conducted on the original lysates (infectivities of 100% for MpoV-45T, 83% for MpV-08T and 72% for PgV-07T) and after UV exposure (within 24 h, stored at 3 °C). Virus cultures tested had total abundances between 5.6 and 9.1 × 10^6^ viruses mL^−1^ (counted by flow cytometry). Viral infectivity of non-UV treated controls was set to 100% to allow comparison between the different viruses. Experiments were conducted in triplicate and pooled for the end-point dilution assay.

### 2.7. Statistical Analyses

Statistical analyses were performed using R [69]. UV-AB experiments 1 and 2 were combined to have more replicates for the 48 h UV-AB exposure treatment and PAR-control after finding no effect of experiment (using the ARTool package [70]). Kruskal–Wallis in combination with Dunn’s post-hoc tests [71] were used after checking for normality of the data and homogeneity of variances.

## 3. Results

### 3.1. UV Effect on Algal Host and Virus

**Figure 1:**
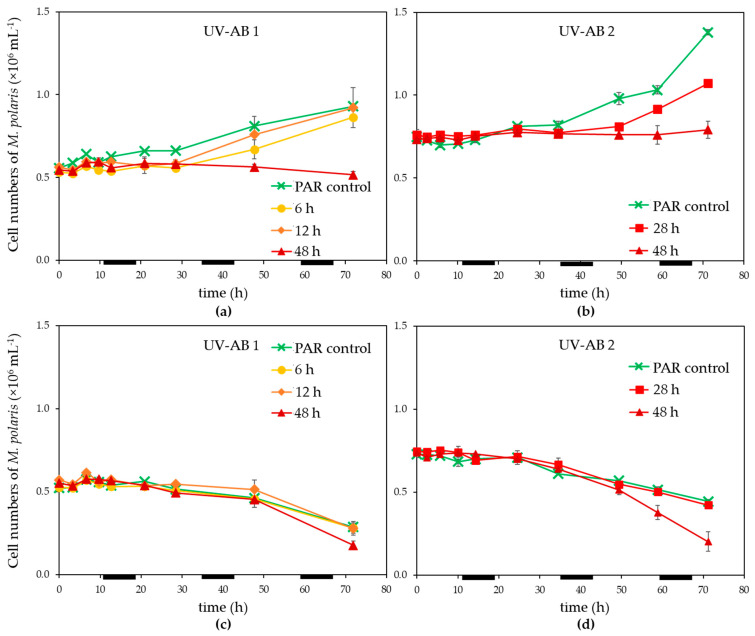
Temporal dynamics of (**a**,**b**) non-infected controls, (**c**,**d**) infected *Micromonas polaris* abundance under PAR + UV-AB exposure (6, 12, 28 and 48 h, over 72 h experimental duration) compared with PAR-only controls for (**a**,**c**) experiment 1 and (**b**,**d**) for experiment 2. Average values with standard deviation (*n* = 2, except for experiment 2 PAR-only control where *n* = 1). Light–dark cycle was 16:8 h, during dark periods UV exposure was stopped. The horizontal black bars indicate the dark period.

Exposure of *Micromonas polaris* to UV-AB up to 28 h led to an initial reduction of the growth of *M. polaris* (in contrast to the PAR-only control; Figure 1a,b), however, growth resumed when UV-AB exposure was stopped. The growth rates for *M. polaris* during recovery from 6 to 28 h UV-AB radiation were with 0.16–0.31 d^−1^ which was largely comparable to the 0.21 ± 0.04 (*n* = 3) of the PAR-only controls. Continuous UV-AB exposure during the light period (48 h total UV radiation during the 72 h experiment) resulted in halted growth (between 0 and 0.02 d^−1^; significantly different to growth rate of PAR-controls, *p* = 0.049). When running experiment 2 longer (154 h), growth resumed (0.08 d^−1^). Virus infection resulted in a decline of *M. polaris* abundances around 24 h post infection (p.i.), independent of the UV-exposure, except that the 48 h UV-AB radiation showed a larger reduction (Figure 1c,d).

**Figure 2:**
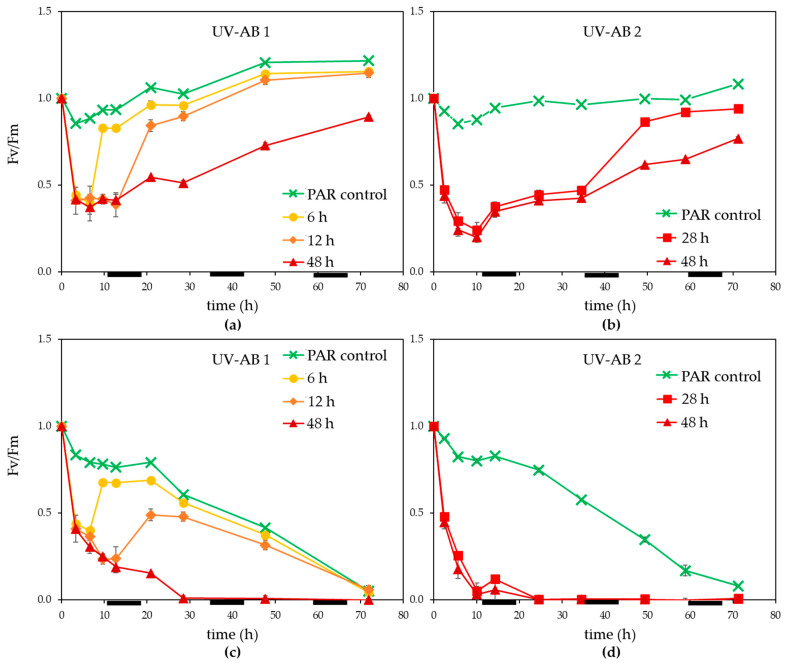
Photosynthetic efficiency (Fv/Fm., normalised to starting value) for (**a**,**b**) non-infected controls, (**c**,**d**) infected *M. polaris* under UV-AB exposure during (**a**,**c**) experiment 1 and (**b**,**d**) experiment 2. Average values with standard deviation (*n* = 2 except for experiment 2 PAR-only control where *n* = 1). The horizontal black bars indicate the dark period.

Within 3 h of UV exposure Fv/Fm dropped more than 50% (Figure 2a,b). Once UV exposure stopped, Fv/Fm recovered steadily for non-infected controls, the time to recovery (between 3 and 12 h) depending on the duration of UV. The 48 h UV treatment displayed only a partial recovery of Fv/Fm during the 72 h experiment (significant difference at the end of the experiment between PAR control and 48 h UV-AB non-infected treatment, *p* = 0.002). Viral infection caused Fv/Fm to decline in general and especially for the UV treated cultures (Figure 2b,c). For the 6 and 12 h UV-AB exposed MpoV-45T infected cultures, a partial recovery of Fv/Fm was observed once the UV-AB exposure stopped. This was not the case for the 28 and 48 h UV-AB exposed infected cultures.

**Figure 3:**
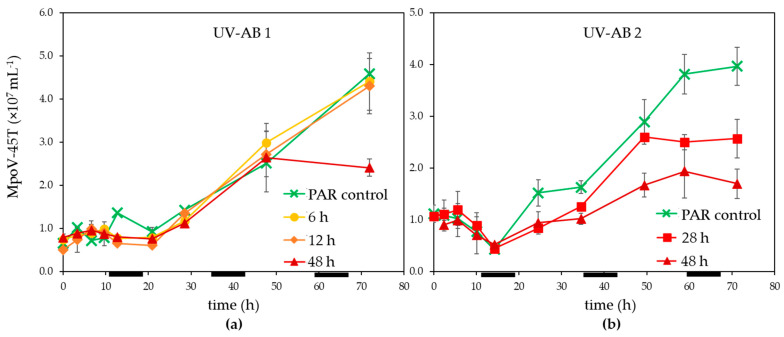
Temporal abundance of virus MpoV-45T under UV-AB exposure during (**a**) experiment 1 and (**b**) experiment 2. Average values with standard deviation (*n* = 2 except for experiment 2 PAR-only control where *n* = 1). The horizontal black bars indicate the dark period.

Infected *M. polaris* cultures started declining about 24 h post infection (p.i.), independent of the UV-exposure except that the 48 h UV-AB treatment showed a larger reduction (Figure 1c,d). The time until the release of the progeny viruses (latent period) was not affected by the UV-AB treatment. There was some variation between the two experiments (Figure 3) but still within the published latent period for this virus–host model system [42,52] and similar for UV-treated and PAR-only controls.

The virus production rates (mL^−1^ h^−1^), calculated after 21–25 h p.i. until the end of the experiments, were reduced for the 28 and 48 h UV-AB exposure to respectively 71 and 31–45% of PAR-only controls (28 h UV treatment not significantly different to PAR control, 48 h UV treatment *p* = 0.047). The MpoV production rates for the shorter UV-AB exposures (6 and 12 h) were unaffected (99 and 101% of PAR-only controls, Table 1).

For UV-AB experiment 2 the original sampling was conducted until 96 h p.i. and the MpoV-45T virus data showed that the virus abundances levelled off after 72 p.i. This implied that the viral burst size (the number of viruses released per lysed host cell) could be estimated for this experiment. The lower production rates for 28 and 48 h UV-AB exposure resulted in strongly reduced burst sizes, only 53% (64) and 23% (27) of the PAR-only control (average burst size 120 ± 14).

### 3.2. UV-C Treatment

The effect of seven UV-C radiation doses (0, 25, 50, 100, 200, 400, and 800 mJ cm^−2^) was tested on the infectivity of three different algal viruses: the two *Micromonas* viruses (one polar MpoV-45T and one temperate MpV-08T) and PgV-07T infecting the temperate *Phaeocystis globosa*. The polar MpoV and the temperate PgV both displayed a drop of more than 95% infectivity with a UV-C dose of only 25 mJ cm^−2^ (Table 2), with PgV being the most sensitive virus. A log^−4^ reduction (99.99%, to comply to BW legislation) for PgV was therefore already reached after 100–200 mJ cm^−2^ UV-C, as compared with a dose above 200 mJ cm^−2^ for MpoV. Complete loss of infectivity was observed after 200 and 400 mJ cm^−2^ for PgV and MpoV, respectively. The other *Micromononas* virus MpV was the least sensitive virus, needing a dose of up to 400 and 800 mJ cm^−2^ for the log^−4^ reduction and total loss of infectivity (Table 2). The UV-C treatment did not affect the virus abundances, independent of UV-C dose (after treatment similar to start concentration before UV-C radiation).

## 4. Discussion

### 4.1. Effects of UV-AB on Virus-Host Interactions

During the time of UV exposure, algal growth was negatively affected, but regained once UV radiation stopped. For the 6, 12, and 28 h UV treatments, algal net growth rates again reached comparable rates as the PAR-only controls (any reduction in algal abundance at the end of the experiment, compared with the PAR-only control, was non-significant). UV-B radiation can lead to direct inhibition of DNA synthesis; thus, the lowering of virus production might be explained by inhibition of DNA synthesis and gene transcription in UV-AB treatments. Moreover, UV-B radiation can result in the production of, e.g., cyclobutyl pyrimidine dimers (CPDs) and pyrimidine (6-4) pyrimidinone (6-4 PD) [72]. Picophytoplankton seems to be vulnerable to CPD accumulation, affecting the internal algal reproduction machinery [73] and likely the host’s ability to produce viruses. Photo-enzymatic DNA repair uses light in the 300–500 nm range to stimulate the reversal of the CPD-protein complex (after binding of proteins to CPD), however picophytoplankton are reported to have a low potential for this [9]. Dark DNA repair may still have taken place during the daily 8 h dark period [72,73], which would have reduced possible detrimental UV effects for the virus proliferation in the shorter (6 and 12 h) UV exposed cultures.

For bacteria it is reported that growth rate directly relates to virus production [74,75], however studies using phytoplankton–virus model systems have shown that the metabolic state of the host may be more important than the actual growth rate (e.g., [53,76]). Environmental conditions affecting algal host metabolism and cellular stress such as light intensity and nutrient availability were found to influence virus latent period, burst size and even infectivity [42,77,78]. A decrease in virus production can be seen as an indicator of algal host stress [79], and in the current study the distinct response of vital physiological (strongly reduced Fv/Fm) cellular conditions to UV-AB exposure are indeed indicators of stress. Rapid decrease in Fv/Fm and swift recovery following UV stress release have been documented for phytoplankton [80,81,82]. Photoinhibitory stress results in a decrease in the maximum photochemical quantum yield of photosystem II and the relatively fast recovery of the 6 and 12 h UV-AB exposed cultures implies a dynamic recovery whereas the exposure to 28 and 48 h involves more chronic damage. The relatively short UV-AB exposed cultures (6 and 12 h) were actually able to recover their photosynthetic capacity in time for MpoV release from the host cell. Their virus production rates were comparable to the PAR-only control, yet for the 28 and 48 h UV-AB treated cultures the strong drop in Fv/Fm could not be restored and likely contributed to the reduced virus production rate and burst size. In the case where the photosystems in a primary producer host (generating energy) are not working efficiently, the production of a high viral yield becomes eventually diminished [83]. Note that UV exposure may also induce biochemical cellular stress by elevated reactive oxygen species (ROS) production in algal cells [84,85]. Formation of ROS may aid to a reduced virus production through DNA damage [72] and negatively affecting RuBisCO and photosynthetic pigments (electron transport).

UV-A and UV-B specific effects on the Arctic *M. polaris* were examined by using a LEE-130 clear filter (LEE Filters, Hampshire, UK) and the UV-A only exposure did not negatively affect the growth of the 48 h UV treated *M. polaris* to the extent of the UV-AB experiments (Figure S1). The reduced growth in the UV-AB experiments was thus mainly caused by UV-B. The small reduction under 48 h UV-A may even have been induced by some (unwanted) leftover UV-B radiation as the used filter did not adsorb wavelengths 312 to 315 nm (6.5% of the total emitted UV-B radiation), allowing for possible false positive biological effects. The photosynthetic capacity Fv/Fm dropped in response to UV-A exposure equally as to UV-AB (Figure S1), alluding UV-A as the cause of this outcome. This fits with results of Boucher and Prézlin [86], who found a higher photoinhibition of diatoms in the Southern Ocean due to UV-A, than to UV-B. An additional pilot testing MpoV-45T proliferation under only UV-A radiation (6, 12, 28 and 48 h) gave largely similar results as under UV-AB, but in this case only the 54 h UV-A treatment caused a reduced virus production rate (70% of PAR-only control). These results imply that both UV-A- and UV-B-induced stress of the host cells resulted in reduced MpoV-45T production.

Jacquet and Bratbak [34] also used a *Micromonas* host–virus model system and found no effect of UV-B on virus abundance dynamics or infectivity but did report a high sensitivity of the algal host (died rapidly) to short UV-B exposure (0.22 W m^−2^, 4 h daily for 4 days). The total dose used was two-fold higher than for this study’s UV-AB experiments (72 h), however, experiment 2 ran longer (154 h) and even then, a decrease in algal abundances was not found over time. Their use of *M. pusilla* occurring in temperate waters, in contrast to the polar *M. polaris*, may help explain the difference in growth response. Many primary producers synthesise UV absorbing substances known as mycosporine-like amino acids (MAA) [87]. Polar phytoplankton in general contain more MAAs (therefore are able to recover faster from UV exposure effects [88]), and additionally, picophytoplankton seem to be relatively resistant to photosynthetic inhibition compared with larger phytoplankters [73]. As polar phytoplankton are more exposed to UV-radiation in general (ozone hole, 24 h light in summer), a higher resistance to UV-radiation and efficient photo-repair system is beneficial for polar phytoplankters. However, the low temperatures in polar waters may lead to a decreased repair efficiency [20,24]. With global warming it could be that repair efficiency improves, thus (partially) counteracting predicted increases in UV-induced damage in the future.

### 4.2. Ecological Relevance

This study revealed that UV radiation had a negative impact on MpoV-45T production only when the host was exposed for prolonged periods of UV-AB (>28 h exposure over a 72 h experiment). Intensified vertical stratification from global climate change is likely to promote such circumstances. The intensities used in this study represented natural conditions at the mid-mixed layer during a sunny day. As such they represent an average of what vertically mixed phytoplankton cells experience, however, to what extent short periods of higher (surface waters) and lower (bottom mixed layer) UV-AB radiation during well-mixed conditions influence the host–virus interactions needs to be examined in more detail.

Polvani and colleagues [89] concluded that ozone depleting substances contribute largely to recent Arctic warming. With the ongoing warming it is likely that more coloured dissolved organic matter (cDOM) will enter the coastal Arctic seas due to the thawing of terrestrial permafrost regions [90]. This will lead to shading from UV radiation but will also cause shading from PAR, thus resulting in both a positive and a negative impact on phytoplankton [20]. For both the Arctic and Antarctic, melting sea ice and reduced snow coverage will lead to increased UV radiation in the surface ocean, however the effect might be stronger in Antarctic waters, due to the absence of cDOM.

The ozone hole over the Arctic was bigger than ever in 2020 [15] but has closed after the polar vortex split, allowing ozone-rich air into the Arctic. Recently, studies [91,92] postulated that conditions favourable for sizable, winter ozone loss could persist in the Arctic or even worsen until the end of this century. At the same time, Antarctica still faces a giant ozone hole and assuming these findings can be extrapolated to the other polar region (Antarctic seas), the influence of UV-AB on phytoplankton virus production and consequently host population dynamics is only growing.

### 4.3. Viral Inactivation by UV-C

Ballast water treatment must often achieve 99.99% (log^−4^) removal of organisms [93], but there are no guidelines for viruses in the latest Guidelines for Approval of Ballast Water Management Systems [94]. For drinking water, a UV-C dose of 40 mJ cm^−2^ is suggested (NSF International 2009 [95]), which this study shows is not nearly enough to inactivate the tested marine algal viruses. A BW UV-treatment system can apply maximum doses of 300 mJ cm^−2^ [65], but this was still not enough to cause a log^−4^ reduction in infectious viral entities of the temperate *Micromonas* virus MpV (800 mJ cm^−2^ was required instead). Complete virus inactivation was only established at 200, 400 and 800 mJ cm^−2^ for PgV, MpoV and MpV, respectively. These doses are higher compared with a study testing 4 different bacteriophages in BW where a log^−4^ reduction in infectious titer was reached after a UV-C dose of 6.4 to 64 mJ cm^−2^ [96]. Two studies on natural dsDNA viruses [97,98] found that 100 to 220 mJ cm^−2^ was enough to inactivate the viruses by log^−3^ (99.9%). The doses needed to obtain a log^−3^ reduction in infectious MpoV and PgV (100 mJ cm^−2^) matched well with these reports. The dose needed for MpV was, however, still higher (400 mJ cm^−2^).

The differences in viral tolerance to UV-C radiation can likely be explained by the virus genome characteristics and particle structure [99]. Larger sized genomes seem easier to inactivate due to their larger surface area and consequently higher likelihood that DNA damage will occur [98]. As the genome size of MpoV, MpV and PgV were 205, 208, and 466 kb, respectively [52,61,64], PgV should be the most sensitive to UV-C radiation. This was indeed the case with a log^−3^ (99.9%) reduction at 100 mJ cm^−2^ and full inactivation at 200 mJ cm^−2^.

Secondly, viruses with AT-rich genomes and thus higher potential dimer (T-T) sites have a higher potential for UV damage, which increases the change of reduced infectivity [99]. The AT% of related and similarly large genome viruses as PgV-07T is 68% [100], and for related MpVs it varies between 57 and 60% [101]. The higher AT% for PgV corresponds indeed with the highest sensitivity for UV-C. MpV being less sensitive to UV-C than MpoV may then indicate that it has a lower AT% than MpoV; something to still be investigated.

The *Micromonas* viruses have comparable genome sizes and both have a lipid membrane [102], yet they differ strongly in their sensitivity to UV-C. Previous studies report that the Arctic *Micromonas* host forms a separate ecotype adapted to lower temperatures [37,103], which also holds for their viruses. It is unlikely, however, that temperature has an influence on UV-C sensitivity, considering PgV is also a temperate, lipid-containing dsDNA algal virus (that can even co-occur with MpV). The authors of this paper speculate that it is more likely that MpoV and MpV differ in their AT%.

With shipping activity increasing in the Arctic in the coming decades (due to global warming induced ice melt [43,44]), it is of ecological importance to evaluate the current BW procedures for inactivation of (polar) marine viruses. The use of UV-C in ballast water systems (and other water treatment facilities) is still one of the best ballast water treatments [104], however the results indicate that the current UV-C doses given in BW treatment systems would need to be increased above 300 mJ cm^−2^ for full inactivation of natural viruses. In conclusion, it is recommended to use UV-C doses of at least 400 mJ cm^−2^ to inactivate marine viruses by the desired log^−4^ BW legislation for organisms. This is assuming a UV transmittance (UV-T) of the water of 90% or higher. In case of more turbid coastal BW (e.g., port water UV-T ranges from 49 to 95% [105]) even higher UV-C doses or pre-treatments (e.g., chemical or filtrations) should be applied.

## Figures and Tables

**Table 1:** Average virus production rates (*n* = 2, average ± SD except for PAR-only controls (0 h UV) in experiment 1 where *n* = 1).

Experiment	UV-AB Treatment (h)	Virus Production (×10^5^ Viruses mL^−1^ h^−1^)
1	0	7.2
	6	7.1 ± 1.8
	12	7.2 ± 1.1
	48	3.2 ± 0.6
	0	5.2 ± 2.4
2	28	3.7 ± 9.6
	48	1.6 ± 1.1

**Table 2:** Average infectivity of 3 different marine viruses (1 polar, 2 temperate) after different UV-C doses. Infectivity is expressed as % of control. Infectivities at 0 UV-C were 100, 83 and 72% for MpoV-45T, MpV-08T and PgV-07T, respectively. *n* = 5 for PAR-only controls and *n* = 3 for UV-C treatments.

mJ cm^−2^	MpoV-45T	MpV-08T	PgV-07T
0	100	100	100
25	4.69	63.3	0.61
50	2.99	10.0	1.44
100	0.06	25.6	0.02
200	0.01	1.84	0.00
400	0.00	0.06	0.00
800	0.00	0.00	0.00

## Data Availability

The data presented in this study are available on request from the corresponding author.

## Supplementary Materials

The following are available online at https://www.mdpi.com/article/10.3390/microorganisms9122429/s1, Figure S1: Exposure of non-infected *M. polaris* to UV-A radiation; Figure S2: Light spectrum of lamps used to provide PAR; Figure S3: Light spectrum of UV-AB lamps; Table S1: UV-AB intensities and attenuation coefficients (µacc); Table S2: Values used to calculate UV-C exposure times; Table S3: Abundances of *M. polaris*; Table S4: Abundances of MpoV-45T.
