# Supplementary material for: Effects of UV Radiation on the Chlorophyte Micromonas polaris Host–Virus Interactions and MpoV-45T Virus Infectivity
Source: Microorganisms. 2021 Nov 25;9(12):2429. doi: 10.3390/microorganisms9122429 (PMC8705608; doi:10.3390/microorganisms9122429)
Supplement: Supplementary file 1 [file microorganisms-09-02429-s001.zip › microorganisms-1453372-supplementary.pdf]

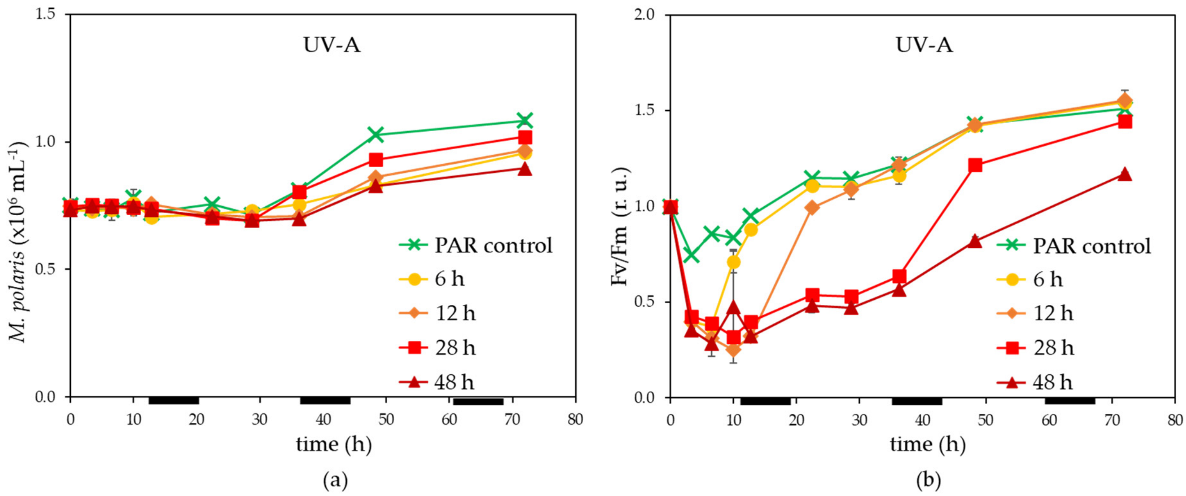

Figure S1: Exposure of non-infected *M. polaris* to UV-A radiation. (a) Phytoplankton abundance dynamics, (b) Photosynthetic efficiency (Fv/Fm, relative units r.u., normalized to starting value). Average values with standard deviation (n = 2 except for PAR-only control and 28 h UV-AB where n = 1). The horizontal black bars indicate the dark period.

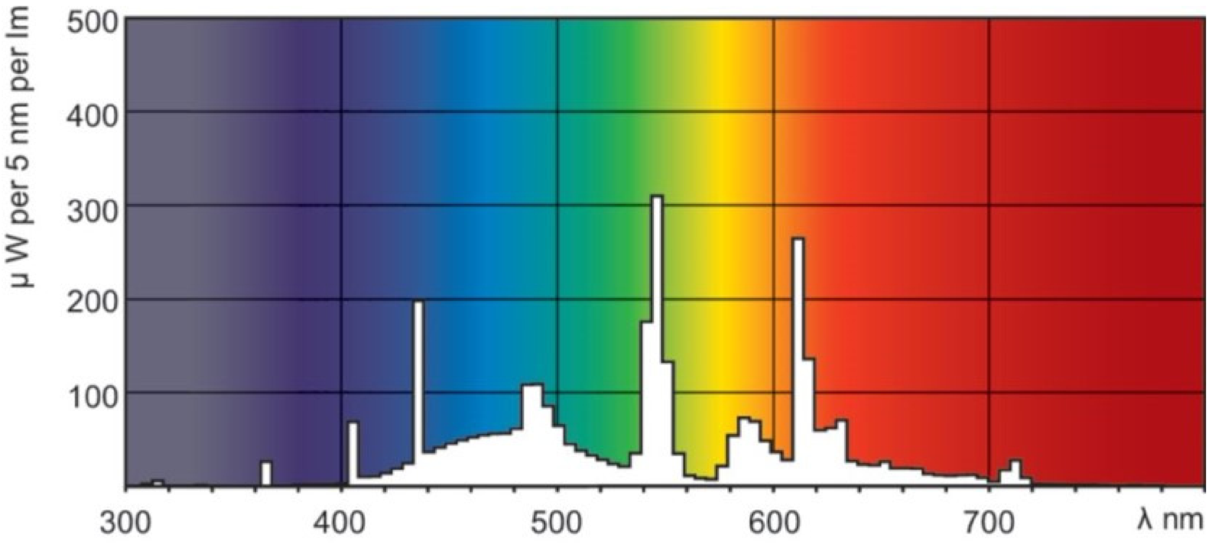

Figure S2: Light spectrum of lamps used to provide PAR.

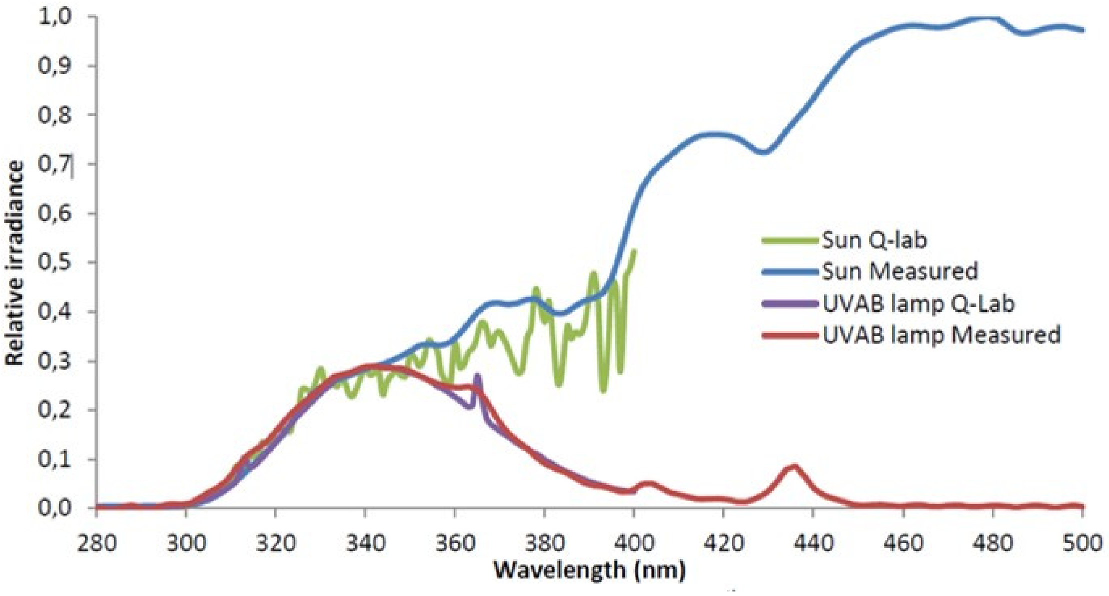

Figure S3: Light spectrum of UV-AB lamps.

Table S1: UV-AB intensities and attenuation coefficients (µac). The averages of these values were used to calculate the average UV-dose received in the Mixed Layer Depth (MLD) of polar regions. Average µac for the Arctic were 0.665 and 0.28 for UV-A and UV-B, respectively, with average UV-intensities (UV-I) of 6.6 and 0.4 each and MLD was up to 30 m. For the Southern Ocean, average µac were 0.38 and 0.28 for UV-A and UV-B, with average intensities of 27.9 and 1.1 for UV-A and UV-B, respectively, with a maximum MLD of 50 m. The UV-intensity at a given depth ($UV - I_D$) was calculated using the following formula following formula: $UV - I_D = UV - I * e^{n*(-\mu ac)}$, with n being the depth in meter in steps of 1. The resulting values were averaged over the MLD for each region to get to the average UV-Intensity within the mixed layer.

	UV-Intensities			Attenuation Coefficients		
	UV-A (W m^{-2})	UV-B (W m^{-2})	reference	UV-A (m^{-1})	UV-B (m^{-1})	reference
Arctic	0.38 - 0.47	0.015 - 0.0189	[1]	0.39 - 0.94	0.225	[2]
	19	1.09	[3]		0.34	[4]
Southern Ocean		1.8	[2]			
	35	0.9	[5]	0.19 - 1.1		[2]
	20.8	0.5	[6]	0.157 - 0.262	0.284	[7]

Table S2: Values needed to calculate UV-C exposure times: Three phytoplankton viruses were exposed to UV-C doses between 0 – 800 mJ cm^{-2}. UV-C doses were controlled by exposure time. Needed exposure time was calculated following [8] using the following values:

Solution volume	15 mL
Water path length	0.63 cm
Distance UV-lamp to water surface	48.6 cm
Divergence Factor	0.9872
Absorption Coefficient	0.008 cm^{-1}
Total Absorbance	0.001
Water Factor	0.9988
Petri Factor	0.818 – 0.832 mW cm^{-2}
Reflection Factor	0.975
Divergence Factor	0.986
Resulting average germicidal irradiance throughout the water column	0.644 – 0.666 mW cm^{-2}

Table S3: Abundances of *Micromonas polaris* (x10^5 mL^{-1}) for cultures without viruses added (C) and with MpoV-45T added (V), exposed to PAR-only (controls) or UV-AB for total duration of 6, 12, 28 and 48 h for experiment 1 and experiment 2 (*n* = 2, except for Exp. 1 PAR-only controls where *n* = 1).

**Exp. 1**	**PAR-only**		**6 h**				**12 h**				**48 h**			
**Time (h)**	**C 1**	**V 1**	**C 1**	**C 2**	**V 1**	**I 2**	**C 1**	**C 2**	**V 1**	**V 2**	**C 1**	**C 2**	**V 1**	**V 2**
**0.0**	5.6	5.2	5.3	5.4	5.2	5.3	5.6	5.6	5.8	5.6	5.4	5.5	5.5	5.6
**3.3**	5.9	5.2	5.2	5.3	5.3	5.1	5.6	5.4	5.5	5.4	5.3	5.5	5.3	5.4
**6.6**	6.4	5.7	5.7	5.6	5.8	5.6	6.0	5.9	6.1	6.2	5.9	5.9	5.8	5.8
**9.7**	5.9	5.6	5.6	5.3	5.5	5.4	5.9	5.9	5.8	5.4	6.1	5.8	5.7	5.9
**12.7**	6.3	5.4	5.3	5.5	5.2	5.4	6.2	5.7	5.8	5.7	5.5	5.6	5.8	5.6
**20.9**	6.6	5.6	5.4	6.0	5.2	5.5	5.8	5.6	5.2	5.5	5.6	6.1	5.3	5.5
**28.5**	6.6	5.2	5.6	5.5	5.2	4.9	6.0	5.7	5.5	5.4	5.8	5.9	4.8	5.1
**47.7**	8.1	4.6	6.3	7.1	4.2	4.9	6.8	8.4	5.5	4.7	5.5	5.8	4.6	4.5
**71.9**	9.3	2.9	8.2	9.1	2.6	3.0	8.4	10.1	3.1	2.5	5.0	5.3	1.9	1.6

**Exp. 2**	**PAR-only**				**28 h UV**				**48 h UV**			
**Time (h)**	**C 1**	**C 2**	**V 1**	**V 2**	**C 1**	**C 2**	**V 1**	**V 2**	**C 1**	**C 2**	**V 1**	**V 2**
**0**	7.8	7.2	7.2	7.4	7.5	7.7	7.5	7.4	7.3	7.3	7.4	7.4
**2**	7.3	7.2	7.3	7.2	7.5	7.5	7.5	7.3	7.2	7.6	7.2	7.0
**6**	7.1	6.9	7.2	7.2	7.5	7.7	7.6	7.5	7.6	7.3	7.3	7.3
**10**	7.1	7.0	7.0	6.6	7.4	7.6	7.4	7.4	7.4	7.2	7.6	7.1
**14**	7.4	7.2	7.2	6.8	7.6	7.6	7.1	6.8	7.5	7.6	7.3	7.3
**25**	8.0	8.2	7.4	6.8	7.8	8.1	7.2	7.1	7.8	7.7	7.1	6.9
**35**	8.0	8.4	6.1		7.7	7.7	6.9	6.4	7.6	7.7	6.4	6.4
**49**	10.0	9.5		5.7	8.1	8.1	5.3	5.6	7.4	7.8	5.4	4.9
**59**	10.5	10.1	5.3	5.0	9.1	9.2	5.2	4.9	7.2	8.0	4.1	3.5
**71**	13.7	13.9	4.5	4.4	10.6	10.8	4.4	4.1	7.6	8.3	2.4	1.6

Table S4: Abundances of MpoV-45T (x10^{6} mL^{-1}) from infected *Micromonas polaris* cultures exposed to PAR-only (controls) or UV-AB for total duration of 6, 12, 28 and 48 h for Experiment 1 and Experiment 2 (n = 2, except for Exp. 1 PAR-only controls, there n = 1). Due to technical failure no values were obtained for Exp. 2, 48 h treatment, T0.

Exp. 1	PAR-only	6 h		12 h		48 h	
**Time (h)**	**V 1**	**V 1**	**V 2**	**V 1**	**V 2**	**V 1**	**V 2**
**0**	6.6	7.7	7.1	5.1	5.1	7.3	8.5
**3**	10.2	8.5	8.3	9.4	5.3	8.4	9.2
**7**	7.2	7.5	10.3	11.2	8.5	8.6	10.4
**10**	7.8	0.0	9.9	9.8	8.1	10.7	6.8
**13**	13.6	8.3	7.1	0.0	6.5	8.1	7.9
**21**	9.2	6.2	9.5	5.6	6.6	6.8	8.4
**28**	14.2	12.6	10.9	14.0	13.0	10.7	11.7
**48**	25.1	31.8	28.0	30.9	23.5	20.8	32.0
**72**	45.9	48.7	39.4	38.5	47.5	25.5	22.7

Exp. 2	PAR-only		28 h UV		48 h UV	
**Time (h)**	**V 1**	**V 2**	**V 1**	**V 2**	**V 1**	**V 2**
**0**	12.4	10.0	10.8	10.4		
**2**	8.7	13.0	10.3	11.9	9.7	8.4
**6**	11.2	9.3	14.5	9.4	12.2	7.7
**10**	7.4	8.1	7.1	10.7	9.5	4.5
**14**	4.8	3.7	4.4	4.5	5.0	5.4
**25**	17.0	13.4	8.9	7.9	7.9	10.9
**35**	15.4	17.2	13.1	12.1	11.0	9.4
**49**	25.8	32.0	26.1	25.9	18.3	15.1
**59**	40.9	35.4	26.1	24.0	23.0	15.8
**71**	42.2	37.1	23.1	28.3	19.0	14.9

References

1. Lakkala, K.; Jaros, A.; Aurela, M.; Tuovinen, J.P.; Kivi, R.; Suokanerva, H.; Karhu, J.M.; Laurila, T. Radiation measurements at the Pallas-Sodankylä Global Atmosphere Watch Station — diurnal and seasonal cycles of ultraviolet, global and photosynthetically-active radiation. *Boreal Environ. Res.* **2016**, *21*, 427–444.
2. Hoyer, K. Occurrence, induction and physiological importance of UV-absorbing substances in polar macroalgae= Vorkommen, Induktion und physiologische Bedeutung UV-absorbierender Substanzen in polaren Makroalgen. *Berichte zur Polar-und Meeresforschung= Reports polar Mar. Res.* **2003**, *440*, 155.
3. Bischof, K.; Hanelt, D.; Tüg, H.; Karsten, U.; Brouwer, P.E.M.; Wiencke, C. Acclimation of brown algal photosynthesis to ultraviolet radiation in Arctic coastal waters (Spitsbergen, Norway). *Polar Biol.* **1998**, *20*, 388–395, doi:10.1007/s003000050319.
4. Hanelt, D.; Tüg, H.; Bischof, K.; Groß, C.; Lippert, H.; Sawall, T.; Wiencke, C. Light regime in an Arctic fjord: A study related to stratospheric ozone depletion as a basis for determination of UV effects on algal growth. *Mar. Biol.* **2001**, *138*, 649–658, doi:10.1007/s002270000481.
5. Buma, A.G.J.; De Boer, M.K.; Boelen, P. Depth distributions of DNA damage in antarctic marine phyto and bacterioplankton exposed to summertime UV radiation. *J. Phycol.* **2001**, *37*, 200–208, doi:10.1046/j.1529-8817.2001.037002200.x.
6. Hughes, K.A. Influence of seasonal environmental variables on the distribution of presumptive fecal coliforms around an Antarctic research station. *Appl. Environ. Microbiol.* **2003**, *69*, 4884–4891, doi:10.1128/AEM.69.8.4884-4891.2003.
7. Figueroa, F.L. Bio-optical characteristics of Gerlache and Bransfield Strait waters during an Antarctic summer cruise. *Deep. Res. Part II Top. Stud. Oceanogr.* **2002**, *49*, 675–691, doi:10.1016/S0967-0645(01)00118-7.
8. Bolton, J.R.; Linden, K.G. Standardization of Methods for Fluence (UV Dose) Determination in Bench-Scale UV Experiments. *J. Environ. Eng.* **2003**, *129*, 209–215.
